# A Mutation in* IL4RA* Is Associated with the Degree of Pathology in Human TB Patients

**DOI:** 10.1155/2016/4245028

**Published:** 2016-02-10

**Authors:** Christoph Hölscher, Lisa Heitmann, Ellis Owusu-Dabo, Rolf D. Horstmann, Christian G. Meyer, Stefan Ehlers, Thorsten Thye

**Affiliations:** ^1^Infection Immunology, Research Center Borstel, Parkallee 22, 23845 Borstel, Germany; ^2^German Center for Infection Research, Germany; ^3^Department of Community Health, College of Health Sciences, Kwame Nkrumah University of Science and Technology, Kumasi, Ghana; ^4^Department of Molecular Medicine, Bernhard Nocht Institute for Tropical Medicine, Bernhard-Nocht-Strasse 74, 20359 Hamburg, Germany; ^5^Microbial Inflammation Research, Research Center Borstel, Parkallee 22, 23845 Borstel, Germany; ^6^Molecular Inflammation Medicine, Christian Albrechts University, Christian-Albrechts-Platz 4, 24098 Kiel, Germany

## Abstract

The contribution of interleukin- (IL-) 4 receptor-alpha- (R*α*-) dependent events in the pathogenesis of tuberculosis (TB) is controversial. We have recently shown IL-13 overexpression in mice to cause recrudescent* Mtb* replication and centrally necrotizing granulomas strongly resembling pathology of human TB. A deletion of IL-4R*α* completely abrogates TB tissue pathology in these mice. To validate our results in human TB patients, we here determined the association of distinct variants of the* IL4*,* IL13*,* IL4RA*,* IL13RA1*, and* IL13RA2* genes with cavity formation in a large Ghanaian cohort of HIV-negative individuals with newly diagnosed pulmonary TB. In fact, the structural variant of the* IL4RA* I50V, previously shown to result in enhanced signal transduction, was significantly associated with greater cavity size, and a variant of* IL13RA2* was associated with disease in females. To evaluate whether the human-like TB pathology in IL-13-overexpressing mice is specifically mediated through the IL-4R*α* subunit, we analyzed IL-13 transgenic mice with a genetic ablation of the IL-4R*α*. In these mice, the IL-13-mediated increased susceptibility, human-like pathology of collagen deposition around centrally necrotizing granulomas, and alternative macrophage activation were abolished. Together, our genetic association study in human TB patients further supports the assumption that IL-13/IL-4R*α*-dependent mechanisms are involved in mediating tissue pathology of human TB.

## 1. Introduction

Human tuberculosis (TB) is a leading global health threat and still constitutes a major medical challenge [1]. In 2014, the disease caused by* Mycobacterium tuberculosis* (*Mtb*) was responsible for 9.6 million new cases worldwide and 1.5 million deaths annually. TB is a systemic disease that becomes manifest most prominently in the lung [2]. Granuloma formation in mycobacterial infections characterizes the inflammatory tissue responses leading to containment of the pathogen. However, when a persistent* Mtb* reactivates within an initially protective granuloma, disease develops. In humans, this postprimary TB is associated with central granuloma necrosis followed by liquefaction of the caseous center, which erodes into the bronchus leaving a cavity and spreading* Mtb* into the environment. Consequently, granuloma necrosis and cavity formation are not only hallmarks of TB pathology, but also responsible for spreading infection. However, mechanisms leading to this typical tissue pathology during postprimary TB are not understood so far.

In human TB, an increased production of the T helper (TH)2 cytokines interleukin- (IL-) 4 and IL-13 is associated with lung damage [3–6], thus indicating that signals mediated through the common IL-4 receptor-alpha (R*α*) may contribute to tissue pathology in postprimary TB. However, no functional connection between IL-4/IL-13 and the development of granuloma necrosis in TB patients has been made yet. In contrast, in experimental TB we have recently shown that overexpression of IL-13 in* Mtb*-infected mice results in recrudescent mycobacterial growth accompanied by centrally necrotizing granulomas strongly resembling pathology of human TB [7]. Therefore, IL-4R*α*-mediated mechanisms appear to direct reactivation, granuloma necrosis, and cavity formation.

To further support our finding that the IL-4R*α* is centrally involved in TB pathology, we here analyzed gene variants in the IL-4/-13-IL-4R*α* pathway for their association with disease severity in a large Ghanaian TB cohort and continued validating our findings in experimental TB.

## 2. Methods

### 2.1. Study Participants, Genotyping, and Statistics

**Figure 1:**
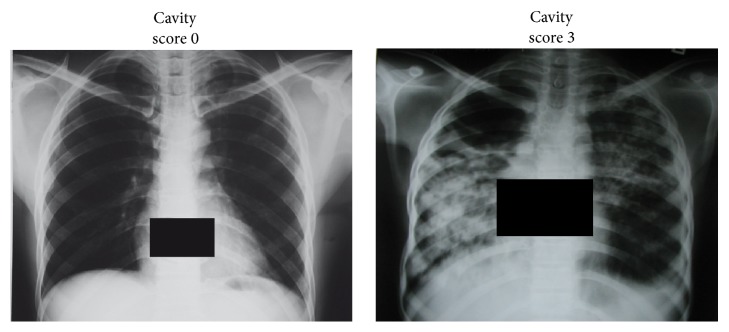
Posterior-anterior chest X-ray of TB cases. Exemplary cavity score 0 (no cavities), cavity score 3 (extended, large cavities) scored blinded by an experienced radiologist, cross-checked by an experienced physician.

Patients with pulmonary TB and healthy control individuals were enrolled in Ghana, West Africa. Further details on the study group and the enrolment procedure of cases and controls are published [8] and described in Supplementary Material, Table S1, available online at http://dx.doi.org/10.1155/2016/4245028. All patients underwent posterior-anterior chest radiography. Pseudonymized films were read by two experienced radiologists. Cavities were individually assigned to the upper right, lower right, upper left, and lower left thoracic quadrants and overall assessed quantitatively being rated “0” (no lesion detectable), “1” (mild lesion), “2” (moderate lesion), and “3” (severe or multiple lesion) (Figure 1).

Genetic variants of the* IL4*,* IL13*,* IL4R*,* IL13RA1*, and* IL13RA2 *were selected due to evidence of association with and functional significance in the TH2-related condition asthma (*IL4-589*, rs2243250 [9];* IL13-1112*, rs1800925 [10];* IL4R* I50V, rs1805010 [11];* IL13RA1*, rs2495636 [12]) and tissue pathology (*IL13RA2*, rs5946040 [13]). Further details on the genotyping protocol and statistical analysis are described in Supplementary Material. Multivariate logistic regression and ordinal logistic regression analyses were performed to determine influences of genetic variants on the disease status (cases* versus* controls) and the severity scores of radiological signs. Calculations included adjustments for age, gender, ethnicity, recruitment centers, and duration of cough, the latter because it was found correlated with the radiographic signs.

### 2.2. Mice

IL-13^tg^, IL-13^tg^  × IL-4R*α* ^−/−^ mice on a BALB/c genetic background were bred under specific-pathogen-free conditions at the Research Center Borstel. All experiments performed were in accordance with the German Animal Protection Law and were approved by the Animal Research Ethics Board of the Ministry of Environment, Kiel, Germany.

### 2.3. Bacteria and Infection of Experimental Mice

*Mtb* (H37Rv) was grown in Middlebrook 7H9 Broth (Difco, Detroit, MI) supplemented with Middlebrook OADC enrichment medium (Life Technologies, Gaithersburg, MI), 0.002% glycerol, and 0.05% Tween 80. Midlog phase cultures were harvested, aliquoted, and frozen at −80°C. After thawing, viable cell counts were determined by plating serial dilutions of the cultures on Middlebrook 7H10 agar plates followed by incubation at 37°C. Before infection of experimental animals, stock solutions of* Mtb* were diluted in sterile distilled water and pulmonary infection was performed using an inhalation exposure system (Glas-Col, Terre-Haute, IN). To infect mice with a low dose of 100 CFU/lung, animals were exposed for 40 min to an aerosol generated by nebulizing approximately 5.5 mL of a suspension containing 10^7^ live bacteria. Inoculum size was checked 24 h after infection by determining the bacterial load in undiluted homogenates of the entire lung of infected mice.

### 2.4. Colony Enumeration Assay and Histology

Bacterial loads in lungs were evaluated at different time points after infection with* Mtb* to follow the course of infection. Organs from sacrificed animals were removed and prepared aseptically, weighed, and homogenized. Tenfold serial dilutions of organ homogenates were plated in duplicate onto Middlebrook 7H10 agar plates containing 10% OADC and incubated at 37°C for 19–21 days. One lung lobe per mouse was fixed in 4% formalin-PBS, set in paraffin blocks, and sectioned (2-3 *μ*m). Histopathological analysis was performed using standard protocols for trichrome staining [14]. Immunohistochemical detection of arginase-1 was performed as previously described [15].

### 2.5. Quantitative Real-Time RT-PCR

Lung samples were isolated before and at different time points after aerosol infection with* Mtb*, weighed, and homogenized in 5 mL of 4 M guanidinium-isothiocyanate buffer. RNA extraction, reverse transcription, and real-time PCR were performed as previously published [16]. Primer and probe combinations selected from the Roche Universal Probe Library were used and are available upon request.

### 2.6. Determination of Arginase Activity

To determine arginase activity in murine tissue, weighed pieces of organs were homogenized in 100 *μ*L of 0.1% Triton X-100 (Sigma) containing a protease inhibitor cocktail (Roche). 50 *μ*L of 10 mM MnCl_2_ (Merck) and 50 mM Tris-HCl (Merck) were added to all samples and the enzyme was activated by heating for 10 min at 55°C. Arginine hydrolysis was conducted by incubating 25 *μ*L of the activated lysate with 25 *μ*L of 0.5 M L-arginine (Merck) at 37°C for 60 min. The reaction was stopped with 400 *μ*L of H_2_SO_4_ (96%)/H_3_PO_4_ (85%)/H_2_O (1/3/7, v/v/v). As a degree of arginase activity, the urea concentration was measured at 540 nm after addition of 25 *μ*L *α*-isonitrosopropiophenone (Sigma; dissolved in 100% ethanol) followed by heating at 95°C for 45 min. One unit of arginase activity is defined as the amount of enzyme that catalyzes the formation of 1 *μ*mol urea/min.

### 2.7. Statistical Analysis of Animal Experiments

Statistical analysis of data obtained in* Mtb*-infected mice was performed by nonparametric ANOVA with Dunn's Multiple Comparison Test.

### 2.8. Online Supplemental Material

The supplementary methods describe the enrolment of the study group. Table S1 illustrates the demographic and Table S2 the radiographic findings within the study group. Table S3 gives the genotype frequencies of variants of TB cases and controls.

## 3. Results and Discussion

### 3.1. In Human TB Patients, a Structural Variant of the Gene Encoding IL-4R*α* Is Associated with Increased Cavity Size or Number

Based on cytokine determinations in TB patients, an IL-4R*α*-mediated TH2 immune response has been implicated in driving TB tissue damage [3]. Particularly, IL-4 levels in peripheral blood mononuclear or bronchoalveolar lavage cells were found increased in TB patients [4, 17–25] and associated with granuloma necrosis and cavity formation [5]. To evaluate whether IL-4-/IL-13-mediated mechanisms are associated with pathology in human TB on a more functional level, we compared the influence of selected genetic variants of the* IL4*,* IL13*,* IL4R*,* IL13RA1*, and the* IL13RA2* genes on the severity of pulmonary lesions in a large Ghanaian cohort of HIV-negative patients with pulmonary TB, comprising a total of 1971 cases and 2332 controls (Tables S1–S3). The frequencies of all genetic variants tested were in Hardy-Weinberg equilibrium among cases and controls. No association of the severity of radiographic findings (Figure 1) was observed with the variants of the* IL4*,* IL13*,* IL13RA1*, and* IL13RA2* genes (Table 1). In striking contrast, the IL4R genotype A/A (I50V), when compared to the genotypes A/G and G/G, occurred more frequently among patients with high radiological severity scores for cavities compared to those with low ones (odds ratio (OR) 1.32, 95% confidence interval (CI) 1.0–1.7, *p* value 0.05). When assuming a recessive mode of inheritance, a similar OR with a higher significance (OR 1.33, CI 1.1–1.7, *p* value 0.016) was evident (Table 1, figures followed by asterisk). The finding indicates that a structural variant of the *α* chain of the IL-4/IL-13 receptor, which was shown to be associated with enhanced signal transduction [11], is associated with large or a greater number of cavities. Statistical calculations to address the question of whether the variants might be of any relevance in a case-control comparison revealed that the* IL13RA2* genotype TT of the variant rs5946040 was, in females, significantly associated with occurrence of disease (OR 1.14, CI 1.1–2.1, *p* value 0.02; Table S3).

Together, by using this genetic approach in human TB we showed for the first time that a single nucleotide polymorphism (SNP) of the* IL4RA* gene, which is known to enhance signal transduction via the IL-4R*α* [11], was significantly associated with increased cavity size in human TB patients. This particular SNP was not associated with overall resistance or susceptibility to TB in the same Ghanaian cohort, but a SNP in the* IL13RA2* gene was weakly associated with disease in females (Table S3, bold figure). Whereas IL-4 mediates its effects through either type I IL-4R (IL-4R*α*/common-gamma chain) or type II IL-4R (IL-4R*α*/IL-13R*α*1), IL-13 has been suggested to execute its IL-4R*α*-dependent effects through type II IL-4R. Nevertheless, IL-13-dependent responses can also develop in the absence of IL-4R*α*-mediated signaling through the IL-13R*α*2 chain [26], which was originally thought to operate exclusively as a decoy receptor for IL-13 [27, 28]. Our genetic association study in human TB patients supports our assumption that IL-13/IL-4R*α*-mediated mechanisms contribute to reactivation and granuloma necrosis in TB. However, because we identified human* IL4RA* and* IL13RA2 *gene variants that are associated with greater risk of cavity development or progression, we could not exclude that in addition to IL-4R*α*- and IL-13R*α*2-mediated signals other mechanisms may also contribute to the pathogenesis of human postprimary TB.

### 3.2. Absence of the IL-4R*α* Abrogates the Increased Susceptibility of* Mtb*-Infected IL-13^tg^ Mice

**Figure 2:**
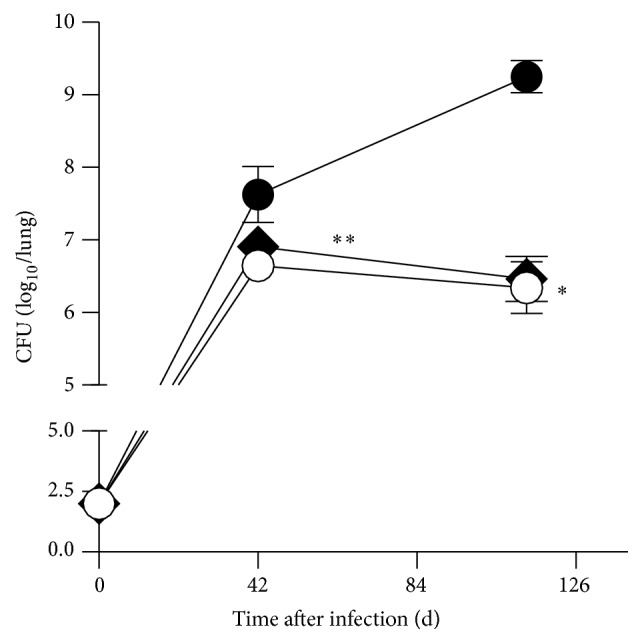
Decreased susceptibility of IL-13^tg^  × IL-4R*α* ^−/−^ mice to* Mtb*. BALB/c (white circles), IL-13^tg^ (black circles), and IL-13^tg^  × IL-4R*α* ^−/−^  (black rhombs) mice were infected with 100 CFU* Mtb *H37Rv per aerosol. At different time points CFU was determined in lung homogenates. Data represent means and standard deviations of 4-5 mice (^*∗∗*^ *p* < 0.01 for comparison of IL-13^tg^ and BALB/c mice; ^#^ *p* < 0.05 for comparison of IL-13^tg^ and IL-13^tg^  × IL-4R*α* ^−/−^ mice; nonparametric ANOVA with Dunn's Multiple Comparison Test). One experiment representative of two performed is shown.

Our human genetic association study revealed a role for both IL-4R*α* and the IL-13R*α*2 in the pathogenesis and progression of TB. Because IL-13-dependent responses are transduced by IL-4R*α*-containing type II IL-4R but may also develop in the absence of IL-4R*α*-mediated signaling through IL-13R*α*2 [26], we next analyzed the specific role of IL-4R*α* in the development of pathology in a murine model. In contrast to humans, after aerosol* Mtb* infection neither wild type nor IL-4R*α* ^−/−^ mice develop granulomas with central necrosis after aerosol* Mtb* infection [7, 29]. Because wild type mice do not appreciably express the IL-4R*α* ligand IL-13 during experimental TB, we have recently established a model in which IL-13 is overexpressed after infection with* Mtb* [7]. In fact,* Mtb*-infected IL-13^tg^ mice are highly susceptible and develop human-like centrally necrotizing granulomas. To analyze the impact of the IL-4R*α* on disease development after* Mtb* infection, we crossed IL-13^tg^ animals with IL-4R*α* ^−/−^ mice [7]. As we have recently published [7], the bacterial loads in the lungs of IL-13^tg^ mice were significantly increased during the course of experimental TB (Figure 2). Deletion of IL-4R*α* abrogates the increased susceptibility of* Mtb*-infected IL-13^tg^ mice (Figure 2). Moreover, we demonstrate an obligatory role for IL-4R*α* in mediating IL-13-dependent progression of experimental TB and we may exclude (also not formally shown) a role for IL-13R*α*2.

### 3.3. Collagen Deposition around Centrally Necrotizing Granulomas Is Abolished in IL-13^tg^ Mice by Genetic Ablation of IL-4R*α*

**Figure 3:**
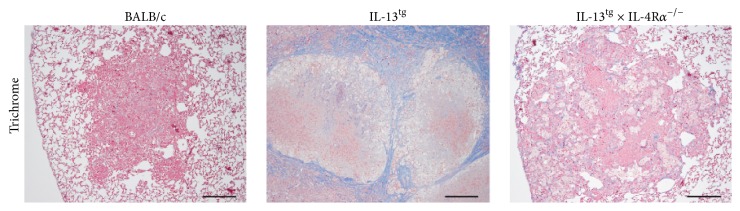
Collagen deposition around centrally necrotizing granulomas is abolished in IL-13^tg^ mice by genetic ablation of the IL-4R*α*. BALB/c, IL-13^tg^, and IL-13^tg^  × IL-4R*α* ^−/−^ mice were infected with 100 CFU* Mtb *H37Rv per aerosol and lung sections stained with trichrome were histologically evaluated after 113 days (*n* = 4-5). Whereas BALB/c mice did not develop necrotic lesions, central granuloma necrosis was found in lungs of IL-13^tg^ mice surrounded by a fibrous rim. Deletion of the IL-4R*α* abolished granuloma necrosis and collagen deposition in IL-13^tg^ mice. Representative photomicrographs of one experiment out of two performed are shown.

After aerosol infection with* Mtb*, circumscript mononuclear foci developed in wild type mice which progressively increased in size over time but never become necrotic [7]. In contrast, IL-13^tg^ mice develop after infection with* Mtb* extensive pulmonary inflammation and centrally necrotizing granulomas and in these mice the deletion of IL-4R*α* prevents granuloma necrosis as shown previously [7]. We also demonstrated that in* Mtb*-infected IL-13-overexpressing animals necrotic granulomas are surrounded by a collagen-rich fibrous layer, resembling human TB lesions [7]. To analyze the contribution of the IL-4R*α* to this typical feature of TB pathology, we here infected BALB/c, IL-13^tg^, and IL-13^tg^  × IL-4R*α* ^−/−^ mice with* Mtb* via the aerosol route and evaluated after 113 days histopathological changes in lung sections stained for collagen (Figure 3). In lungs of* Mtb*-infected BALB/c mice, mononuclear cells accumulated but central granuloma necrosis could not be observed and collagen deposition in lung sections from wild type mice was hardly detectable. As recently published [7], IL-13^tg^ mice developed massive granulomas with a necrotic core consisting of dead and dying cells demarcated by a fibrous capsule-like layer (Figure 3). The absence of the IL-4R*α* completely stopped collagen deposition around centrally necrotizing granulomas (Figure 3). Together, we give further evidence that the IL-4R*α* is involved in promoting key features of TB pathology in a murine model of central granuloma necrosis (and may thereby exclude a contribution of the IL-13R*α*2).

As for our genetic association study in human subjects, we could not exclude that in addition to IL-13/IL-4R*α*-mediated signals other mechanisms also contribute to the development of central granuloma necrosis in* Mtb*-infected mice. Initially, the question why* Mtb*-infected wild type mice do not develop central granuloma necrosis, the typical pathology in human postprimary TB was exciting. Whereas wild type mice do not express appreciable levels of IL-4 and IL-13 after infection with* Mtb*, an increased production of these TH2 cytokines is associated with lung damage in TB patients [3–6]. Consequently,* Mtb* infection of mice that overexpress IL-13 resulted in recrudescent mycobacterial growth accompanied by centrally necrotizing granulomas strongly resembling pathology of human TB [7]. Although our data indicate that the IL-13/IL-4R*α* axis contributes to the development of central granuloma necrosis in TB, other mechanisms involved in the pathogenesis of TB may not be induced in the IL-13 transgenic mouse model. As for TH2 cytokines, these functions may be absent in* Mtb*-infected wild type mice but prominently expressed in human postprimary TB. However, overexpression of these functions still has these intrinsic limitations.

### 3.4. Obliterated Alternative Macrophage Activation in* Mtb*-Infected IL-13^tg^  × IL-4R*α* ^−/−^ Mice

**Figure 4:**
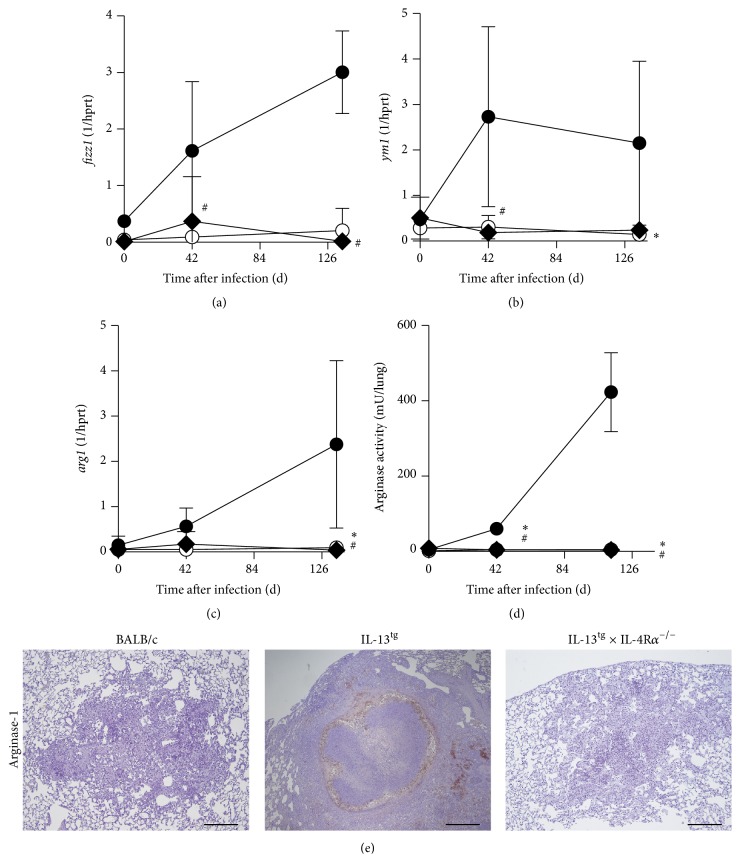
In the absence of the IL-4R*α* alternative macrophage activation is obliterated in* Mtb*-infected IL13^tg^ mice. BALB/c (white circles), IL-13^tg^ (black circles), and IL-13^tg^  × IL-4R*α* ^−/−^ (black rhombs) mice were infected with 100 CFU* Mtb *H37Rv per aerosol. At different time points, gene expression of (a)* fizz1*, (b)* ym1*, (c)* arg1*, and (d) arginase activity in lung homogenates of infected mice was determined. Results represent means and standard deviations of 4-5 mice (^*∗*^ *p* < 0.05 for comparison of IL-13^tg^ and BALB/c mice; ^#^ *p* < 0.05 for comparison of IL-13^tg^ and IL-13^tg^  × IL-4R*α* ^−/−^ mice; nonparametric ANOVA with Dunn's Multiple Comparison Test). One experiment representative of two performed is shown. (e) For immunohistological detection of Arg-1, lung sections were prepared from mice at 113 days after infection (*n* = 4-5). Arg-1 expression was not found in the lungs of* Mtb*-infected BALB/c mice but was prominent in granulomas of IL-13^tg^ mice surrounding the necrotic center. Deletion of the IL-4R*α* abolished Arg-1 expression in lungs of IL-13^tg^ mice. Representative photomicrographs of one experiment out of two performed are shown.

We have recently shown that in* Mtb*-infected IL-13^tg^ mice Arg-1-expressing alternatively activated macrophages (aaM*φ*) are significantly induced and typically surround the necrotic centers of granulomas [7]. Specific elimination of Arg-1 in macrophages enhances antimycobacterial effector mechanisms in macrophages and leads to decreased lung bacterial loads during* Mtb* infection [15, 30]. Moreover, arginase activity contributes also to tissue remodeling and fibrosis in other diseases [31]. Because, in human TB, peripheral blood mononuclear cells from patients with pulmonary disease show higher arginase activity [32] and necrotic lesions are surrounded by fibrotic tissue [2], arginase activity in aaM*φ* may also contribute to TB pathology. To evaluate the effect of the IL-4R*α* on the development of Arg-1 expressing aaM*φ* in IL-13^tg^ mice, we infected BALB/c, IL-13^tg^, and IL-13^tg^  × IL-4R*α* ^−/−^ mice with* Mtb* via the aerosol route and analyzed alternative macrophage activation during the course of experimental TB (Figure 4). Gene expressions of* fizz1* (Figure 4(a)) and* ym1* (Figure 4(b)), two prototype markers for aaM*φ*, were hardly detectable in lung homogenates of wild type mice. As recently shown by us [7],* fizz1* and* ym1* were induced in IL-13^tg^ mice to very high levels during the whole course of infection with* Mtb* (Figures 4(a) and 4(b)). The expression of these two markers for alternative macrophage activation was abolished in* Mtb*-infected IL-13^tg^  × IL-4R*α* ^−/−^ mice. In line with the virtually absent induction of* fizz1* and* ym1*,* arg1* mRNA was hardly detectable in wild type mice. But, in contrast,* arg1* expression was significantly elevated in lungs from infected IL-13^tg^ mice during the course of* Mtb* infection (Figure 4(c)). Importantly, in the absence of the IL-4R*α*,* arg1* gene expression was completely abrogated in lung homogenates of* Mtb*-infected IL-13^tg^ mice (Figure 4(c)). In accordance with these findings, only low arginase activity was detectable in lung homogenates from wild type mice during the course of* Mtb* infection (Figure 4(d)). Whereas in IL-13^tg^ mice arginase activity in lung homogenates increased during the course of infection, the deletion of the IL-4R*α* abolished enzyme activity in these mice (Figure 4(d)). Immunohistochemical staining of lung sections for Arg-1 revealed that Arg-1 expression was undetectable in* Mtb*-infected wild type mice but was very prominent in lung granulomas of IL-13^tg^ mice and necrotic centers were typically surrounded by Arg-1-expressing cells (Figure 4(e)) [7]. The deletion of IL-4R*α* obliterated the expression of Arg-1 within the granulomas of* Mtb*-infected IL-13^tg^ mice (Figure 4(e)). Together, these data show that after infection with* Mtb* alternative macrophage activation and Arg-1 expression in IL-13^tg^ mice depend on the presence of the IL-4R*α* and that IL-4R*α*-mediated downstream mechanisms are putatively involved in TB pathology. This contention was recently supported by a report showing that, in patients with pulmonary TB, lung pathology was associated with increased arginase activity, which was further increased in TB patients coinfected with TH2 cytokine-inducing helminths [33]. Hence, in* Mtb*-infected human subjects, IL-13/IL-4R*α*-induced Arg-1 may undermine macrophage effector responses, mediates collagen deposition and tissue remodeling, and thus contributes to tissue damage in human TB. Further genetic association studies and functional analyses should unravel the putative involvement of Arg-1 in the pathogenesis of human postprimary TB.

## 4. Conclusions

These data support our assumption that IL-4R*α* contributes to the development of central granuloma necrosis in human TB. However, IL-4R*α*-dependent downstream mechanisms remain elusive and more functional analyses are required in the human system. Our animal study in IL-13^tg^  × IL-4R*α* ^−/−^ mice may point at aaM*φ*, which undermine antimycobacterial effector functions in macrophages and promote tissue remodeling and subsequently TB pathology. Further analysis in human TB and in the IL-13^tg^ mouse model may unravel IL-4R*α*-mediated functions involved in the pathogenesis of postprimary TB. More importantly, targeting the IL-4R*α* or downstream mechanisms may constitute an approach to mitigate the development of postprimary TB. Because, for example, inhaling soluble IL-4R (altrakincept) was effective in the treatment of moderate asthma in patients [34], this biological may represent a candidate to medicate TB tissue pathology.

## Supplementary Material

The supplementary methods describe the enrolment of the study group. Table S1 illustrates the demographic and Table S2 the radiographic findings within the study group. Table S3 gives the genotype frequencies of variants of TB cases and controls.

## Figures and Tables

**Table 1:** Genotype frequencies of variants of the *IL4*, *IL4R*, *IL13*, *IL13RA1*, and *IL13RA2* genes in TB cases stratified for the size and number of cavities in chest radiographs^A^.

Gene variant		Cavity score				OR	95% CI	*p*
		0	1	2	3			
*IL4-589 rs2243250*	*N*	79	632	509	186			
CC		63.3%	55.7%	54.2%	57.0%	1		
CT		32.9%	37.7%	38.9%	37.1%	0.96	0.6–1.5	0.85
TT		3.8%	6.6%	6.9%	5.9%	0.89	0.6–1.3	0.58

*IL13-1112 rs1800925*	*N*	81	649	517	189			
CC		29.6%	29.7%	34.0%	28.0%	1		
CT		51.9%	50.4%	49.9%	53.5%	1.00	0.8–1.3	0.97
TT		18.5%	19.9%	16.1%	18.5%	0.86	0.6–1.1	0.31

*IL4R I50V rs1805010*	*N*	82	649	518	191			
GG		28.0%	26.0%	24.1%	25.1%	1		
AG		56.1%	51.5%	51.5%	45.0%	0.99	0.8–1.3	0.92
AA		15.9%	22.5%	24.3%	29.8%	1.32	1.0–1.7	0.05
GG/AG^*∗*^		84.2%^*∗*^	77.5%^*∗*^	75.7%^*∗*^	70.2%^*∗*^	1^*∗*^		
AA^*∗*^		15.9%^*∗*^	22.5%^*∗*^	24.3%^*∗*^	29.8%^*∗*^	1.33^*∗*^	1.1–1.7^*∗*^	0.016^*∗*^

*IL13RA1 rs2495636*	*N*	27	226	140	51			
Female								
AA		88.9%	86.3%	85.7%	84.3%	1		
AG		11.1%	13.7%	14.3%	11.8%	1.17	0.7–2.0	0.55
GG		0.0%	0.0%	0.0%	3.9%			nc

*IL13RA2 rs5946040*	*N*	27	227	140	51			
Female								
GG		48.1%	47.1%	47.9%	31.4%	1		
GT		44.4%	40.1%	43.6%	52.9%	1.26	0.9–1.9	0.24
TT		7.4%	12.8%	8.6%	15.7%	0.90	0.5–1.7	0.74

*IL13RA1 rs2495636*	*N*	57	424	380	137			
Male								
A		91.2%	93.4%	94.2%	90.5%	1		
G		8.8%	6.6%	5.8%	9.5%	1.13	0.7–1.8	0.62

*IL13RA2 rs5946040*	*N*	57	420	375	138			
Male								
G		59.6%	71.0%	69.1%	71.7%	1		
T		40.4%	29.1%	30.9%	28.3%	0.93	0.7–1.2	0.59

^A^Odds ratios (OR) and 95% confidence intervals (CI) were calculated by ordinal logistic regression. nc, not calculable. As the *IL13RA1* and the *IL13RA2* genes are located on the X-chromosome, association testing was performed separately for males and females. Genotype frequencies of IL4R I50V in TB cases stratified for the size and number of cavities assuming a recessive mode of inheritance are followed by asterisk.
