# Supplementary material for: A Mutation in IL4RA Is Associated with the Degree of Pathology in Human TB Patients
Source: Mediators Inflamm. 2016 Feb 10;2016:4245028. doi: 10.1155/2016/4245028 (PMC4764744; doi:10.1155/2016/4245028)
Supplement: Supplementary file 1 — The supplementary methods describe the enrolment of the study group. Table S1 illustrates the demographic and Table S2 the radiographic findings within the study group. Table S3 gives the genotype frequencies of variants of TB cases and controls. [file 4245028.f1.pdf]

SUPPLEMENTARY METHODS

Study group and the enrolment procedure.

Between September 2001 and July 2004, patients with pulmonary TB were enrolled at Korle Bu Teaching Hospital, Accra, Komfo Anokye Teaching Hospital, Kumasi, additional hospitals and polyclinics in Accra and Kumasi and at surrounding district hospitals, all in Ghana, West Africa. Unaffected household contacts of patients and community members served as controls. Participants belonged to the ethnic groups of Akan, Gaa-Adangbe, Ewe, and of immigrants from northern Ghana.

Phenotyping of cases included the documentation on structured questionnaires of major tuberculosis symptoms and medical histories as well as assessment of the HIV-1/2 status (Capillus, Trinity Biotech, Bray, Co Wicklow, Ireland), radiographical findings as detemined by posterior-anterior chest X-ray, the examination of Ziehl-Neelsen stained sputum smears, and culturing of mycobacteria on Loewenstein-Jensen agar. Enrolled were 1971 cases 9 - 60 years of age who were sputum-positive for acid-fast bacilli, had characteristic radiographic lesions, were negative in HIV serology, and had no evidence of alcoholism or other generalized disease.

Disclosure of HIV test results was dependent on the documented willingness of participants to be informed and included for HIV-positive patients their prompt referral to counselling and treatment provided by the Ghanaian AIDS Control Programme.

Phenotyping of healthy control individuals included a medical history, clinical examination, posterior-anterior chest radiography and a PPD skin test (Tuberculin Test PPD Mérieux, bioMérieux, Nürtingen, Germany), performed according to the manufacturer´s recommendations. Enrolled were 2332 controls 6 to 66 years of age. Controls had no history of TB or antimycobacterial treatment and no radiological signs of actual or previous pulmonary TB.

The study protocol had been approved by the Committee on Human Research, Publications and Ethics, School of Medical Sciences, Kwame Nkrumah University of Science and Technology, Kumasi, and the Ethics Committee of the Ghana Health Services, Accra. Venous blood samples were taken only after a detailed explanation of the aims of the study, and consent was obtained by signature or thumb print. HIV-positive patients were excluded from the study.

SUPPLEMENTARY TABLES

Table S1. Demographic findings within the study group

N	**Cases** 1971	**Controls** 2332
Sex [% male]	68.2%	59.2%
Median age (range)	32 (9-60)	30 (6-66)
Ethnicity		
Akan	1262 (64.0%)	1374 (58.9%)
Ewe	142 (7.2%)	219 (9.4%)
Gaa-Adangbe	284 (14.4%)	464 (19.9%)
Northerners[A]	248 (12.6%)	245 (10.5%)
n. a.[B]	35 (1.8%)	30 (1.3%)

[A] Comprising the ethnicities of Dagomba, Sissala, Gonja and Kusasi
[B] Not unambiguously assignable

Table S2. Radiographic findings of TB patients[A]

**Severity score**	**Cavitites**
0	101 (5.6%)
1	810 (44.6%)
2	671 (36.9%)
3	236 (13.0%)

[A] Given are numbers of patients and percentages of the total of n = 1818 for which radiographs were available

Table S3. Genotype frequencies of variants of TB cases and controls[A]

Gene variant	TB cases	controls	OR	95% CI	*p*
IL4 -589 rs2243250	N=1912 (%)	N=2284 (%)			
TT	1059 (55.4)	1268 (55.5)	1		
CT	716 (37.4)	867 (38.0)	0.98	0.9-1.1	0.75
CC	137 (7.2)	149 (6.5)	1.10	0.9-1.4	0.44
IL13 -1112 rs1800925	N=1948	N=2306			
CC	623 (32.0)	735 (31.9)	1		
CT	994 (51.0)	1161 (50.3)	1.03	0.9-1.2	0.68
TT	331 (17.0)	410 (17.8)	0.98	0.8-1.2	0.86
IL4R I50V rs1805010	N=1952	N=2312			
GG	494 (25.3)	609 (26.3)	1		
AG	990 (50.7)	1139 (49.3)	1.05	0.9-1.2	0.52
AA	468 (24.0)	564 (24.4)	0.98	0.8-1.2	0.86
IL13RA1 rs2495636 female	N=623	N=946			
AA	535 (85.9)	838 (88.6)	1		
AG	85 (13.6)	102 (10.8)	1.29	0.9-1.8	0.11
GG	3 (0.5)	6 (0.6)	0.76	0.2-3.1	0.70
IL13RA2 rs5946040 female	N=622	N=942			
GG	266 (42.8)	456 (48.4)	1		
GT	276 (44.4)	398 (42.3)	1.17	0.9-1.5	0.15
TT	80 (12.8)	88 (9.3)	1.49	1.1-2.1	**0.02**
IL13RA1 rs2495636 male	N=1329	N=1370			
A	1238 (93.2)	1288 (94.0)	1		
G	91 (6.8)	82 (6.0)	1.14	0.8-1.6	0.40
IL13RA2 rs5946040 male	N=1323	N=1347			
G	922 (69.7)	951 (70.6)	1		
T	401 (30.3)	396 (29.4)	1.05	0.9-1.2	0.60

[A] Odds ratios (OR) and 95% confidence intervals (CI) were calculated by logistic regression and adjusted for age, sex, ethnicity. OR represent estimates of an additive genetic model. As the *IL13RA1* and the *IL13RA2* genes are located on the X-chromosome, association analyses were calculated separately for males and females.
